# Activation of CDK4 by Tax chemosensitizes p53-mutant cells to DNA damage

**DOI:** 10.1186/1742-4690-11-S1-O44

**Published:** 2014-01-07

**Authors:** O John Semmes, Xin Guo, Valia T Mihaylova, Gary M Kupfer

**Affiliations:** 1 Department of Microbiology and Molecular cell Biology, Eastern Virginia Medical School, Norfolk, VA, USA; 2 The Leroy T. Canoles Jr. Cancer Research Center, Eastern Virginia Medical School, Norfolk, VA, USA; 3 Department of Pediatrics, Yale University, New Haven, CN, USA

## 

Human T cell leukemia virus type 1 (HTLV I) Tax protein has been implicated in cellular transformation via perturbations in genomic stability, transcription, and the cell cycle, and is etiologically linked to the development of adult T cell leukemia and various human neuropathies. It has been established that Tax mediates an increase in cyclin D3-dependent CDK4 activity and we and others have demonstrated that expression of Tax correlates with hyperphosphorylation of retinoblastoma protein and activation of E2F transcription factors. In this study, we explore the relationship between Tax-CDK4 binding and its activations. Although steady-state levels of CDK4 remained unchanged, the expression of Tax resulted in a reduction in the proportion of CDK4 bound in an inhibitory complex with Cyclin D3 and p27. Tax binding and CDK4 activation is mediated through the N terminal 25 amino acids, which we also show is sufficient to confer Tax chemosensitization of p53 mutant cells. The Tax-induced stimulation of CDK4 activity results in increased S phase percentage of cells under hypoxic and starvation stress conditions. A 25 amino acid peptide of Tax markedly stimulates CDK4 kinase activity in vitro and can chemosensitize p53 mutant cells as well. Our studies suggest a mechanism for chemosensitization of p53 mutant cells by exploiting Tax-mediated activation of CDK4.

